# Influence of vitamin D supplementation on bone mineral content, bone turnover markers, and fracture risk in South African schoolchildren: multicenter double-blind randomized placebo-controlled trial (ViDiKids)

**DOI:** 10.1093/jbmr/zjae007

**Published:** 2024-01-10

**Authors:** Keren Middelkoop, Lisa K Micklesfield, Neil Walker, Justine Stewart, Carmen Delport, David A Jolliffe, Amy E Mendham, Anna K Coussens, Averalda van Graan, James Nuttall, Jonathan C Y Tang, William D Fraser, Cyrus Cooper, Nicholas C Harvey, Richard L Hooper, Robert J Wilkinson, Linda-Gail Bekker, Adrian R Martineau

**Affiliations:** Desmond Tutu HIV Centre, Institute of Infectious Diseases & Molecular Medicine, University of Cape Town, Observatory, Cape Town 7925, Western Cape, South Africa; Department of Medicine, University of Cape Town, Observatory, Cape Town 7925, Western Cape, South Africa; Division of Physiological Sciences, Department of Human Biology, Faculty of Health Sciences, Health through Physical Activity, Lifestyle and Sport Research Centre (HPALS), University of Cape Town, Newlands, Cape Town 7700, Western Cape, South Africa; Department of Paediatrics, SAMRC/Wits Developmental Pathways for Health Research Unit, Faculty of Health Sciences, University of the Witwatersrand, Parktown, Johannesburg 2193, Gauteng, South Africa; Wolfson Institute of Population Health, Barts and The London School of Medicine and Dentistry, Queen Mary University of London, London EC1M 6BQ, United Kingdom; Desmond Tutu HIV Centre, Institute of Infectious Diseases & Molecular Medicine, University of Cape Town, Observatory, Cape Town 7925, Western Cape, South Africa; Department of Medicine, University of Cape Town, Observatory, Cape Town 7925, Western Cape, South Africa; Desmond Tutu HIV Centre, Institute of Infectious Diseases & Molecular Medicine, University of Cape Town, Observatory, Cape Town 7925, Western Cape, South Africa; Blizard Institute, Barts and The London School of Medicine and Dentistry, Queen Mary University of London, London E1 2AT, United Kingdom; Division of Physiological Sciences, Department of Human Biology, Faculty of Health Sciences, Health through Physical Activity, Lifestyle and Sport Research Centre (HPALS), University of Cape Town, Newlands, Cape Town 7700, Western Cape, South Africa; Department of Paediatrics, SAMRC/Wits Developmental Pathways for Health Research Unit, Faculty of Health Sciences, University of the Witwatersrand, Parktown, Johannesburg 2193, Gauteng, South Africa; Wellcome Centre for Infectious Diseases Research in Africa, Institute of Infectious Disease and Molecular Medicine, University of Cape Town, Observatory, Cape Town 7925, Western Cape, South Africa; Infectious Diseases and Immune Defence Division, Walter and Eliza Hall Institute of Medical Research, Parkville, Victoria 3052, Australia; Biostatistics Unit, SAFOODS Division, South African Medical Research Council, Tygerberg, Cape Town 7505, Western Cape, South Africa; Division of Human Nutrition, Faculty of Medicine and Health Sciences, Stellenbosch University, Tygerberg, Cape Town 7505, Western Cape, South Africa; Department of Paediatrics and Child Health, Paediatric Infectious Diseases Unit, Red Cross War Memorial Children's Hospital, Rondebosch, Cape Town 7700, Western Cape, South Africa; Norwich Medical School, University of East Anglia, Norwich Research Park, Norwich NR4 7TJ, United Kingdom; Departments of Laboratory Medicine, Clinical Biochemistry and Departments of Diabetes and Endocrinology, Norfolk and Norwich University Hospital NHS Foundation Trust, Norwich NR4 7UY, United Kingdom; Norwich Medical School, University of East Anglia, Norwich Research Park, Norwich NR4 7TJ, United Kingdom; Departments of Laboratory Medicine, Clinical Biochemistry and Departments of Diabetes and Endocrinology, Norfolk and Norwich University Hospital NHS Foundation Trust, Norwich NR4 7UY, United Kingdom; MRC Lifecourse Epidemiology Centre, University of Southampton, Southampton SO16 6YD, United Kingdom; NIHR Southampton Biomedical Research Centre, University of Southampton, Southampton SO16 6YD, United Kingdom; University Hospital Southampton NHS Foundation Trust, Southampton SO16 6YD, United Kingdom; MRC Lifecourse Epidemiology Centre, University of Southampton, Southampton SO16 6YD, United Kingdom; NIHR Southampton Biomedical Research Centre, University of Southampton, Southampton SO16 6YD, United Kingdom; University Hospital Southampton NHS Foundation Trust, Southampton SO16 6YD, United Kingdom; Wolfson Institute of Population Health, Barts and The London School of Medicine and Dentistry, Queen Mary University of London, London EC1M 6BQ, United Kingdom; Wellcome Centre for Infectious Diseases Research in Africa, Institute of Infectious Disease and Molecular Medicine, University of Cape Town, Observatory, Cape Town 7925, Western Cape, South Africa; The Francis Crick Institute, London NW1 1AT, United Kingdom; Imperial College London, London W12 0NN, United Kingdom; Desmond Tutu HIV Centre, Institute of Infectious Diseases & Molecular Medicine, University of Cape Town, Observatory, Cape Town 7925, Western Cape, South Africa; Department of Medicine, University of Cape Town, Observatory, Cape Town 7925, Western Cape, South Africa; Blizard Institute, Barts and The London School of Medicine and Dentistry, Queen Mary University of London, London E1 2AT, United Kingdom

**Keywords:** cholecalciferol, bone mineral content, parathyroid hormone, bone turnover markers, fracture risk

## Abstract

Randomized controlled trials (RCTs) to determine the influence of vitamin D on BMC and fracture risk in children of Black African ancestry are lacking. We conducted a sub-study (*n* = 450) nested within a phase 3 RCT of weekly oral supplementation with 10 000 IU vitamin D_3_ vs placebo for 3 yr in HIV-uninfected Cape Town schoolchildren aged 6–11 yr. Outcomes were BMC at the whole body less head (WBLH) and LS and serum 25-hydroxyvitamin D_3_ (25(OH)D_3_), PTH, alkaline phosphatase, C-terminal telopeptide, and PINP. Incidence of fractures was a secondary outcome of the main trial (*n* = 1682). At baseline, mean serum 25(OH)D_3_ concentration was 70.0 nmol/L (SD 13.5), and 5.8% of participants had serum 25(OH)D_3_ concentrations <50 nmol/L. Among sub-study participants, end-trial serum 25(OH)D_3_ concentrations were higher for participants allocated to vitamin D vs placebo (adjusted mean difference [aMD] 39.9 nmol/L, 95% CI, 36.1 to 43.6) and serum PTH concentrations were lower (aMD −0.55 pmol/L, 95% CI, −0.94 to −0.17). However, no interarm differences were seen for WBLH BMC (aMD −8.0 g, 95% CI, −30.7 to 14.7) or LS BMC (aMD −0.3 g, 95% CI, −1.3 to 0.8) or serum concentrations of bone turnover markers. Fractures were rare among participants in the main trial randomized to vitamin D vs placebo (7/755 vs 10/758 attending at least 1 follow-up; adjusted odds ratio 0.70, 95% CI, 0.27 to 1.85). In conclusion, a 3-yr course of weekly oral vitamin D supplementation elevated serum 25(OH)D_3_ concentrations and suppressed serum PTH concentrations in HIV-uninfected South African schoolchildren of Black African ancestry but did not influence BMC or serum concentrations of bone turnover markers. Fracture incidence was low, limiting power to detect an effect of vitamin D on this outcome.

## Lay Summary

Vitamin D—the “sunshine vitamin”—is essential for helping the body to absorb calcium from the diet, which is laid down in bone to improve its strength. There is a lack of clinical trials testing whether vitamin D supplements can improve bone content of calcium and other minerals, or reduce risk of bone fractures (broken bones) in children of Black African ancestry. We therefore conducted such a study, recruiting 1682 schoolchildren aged 6–11 yr living in Cape Town, South Africa. We found that a weekly dose of 10 000 international units (250 micrograms) of vitamin D_3_, given by mouth for 3 yr, was effective in boosting vitamin D levels in trial participants who received it. However, this did not have any effect on bone content of calcium and other minerals. Relatively few children experienced a broken bone during the study, so we were unable to say with confidence whether or not vitamin D supplements might affect this outcome.

## Introduction

Low BMD and related fractures cause a large and increasing global burden of disability-adjusted life years and mortality.^1^ Osteoporosis in adulthood may have its origins in childhood, which is an important period for optimization of bone mass.^2^ Vitamin D has long been recognized to play a key role in promoting bone mineralization,^3^ and observational studies report associations between low circulating concentrations of 25OHD and increased fracture risk in children.^4^ However, an evidence-based international consensus group has concluded that, although children with radiographically confirmed rickets have an increased risk of fracture, children with simple vitamin D deficiency do not.^5^ A 2017 meta-analysis of aggregate data from randomized controlled trials (RCTs) of vitamin D conducted in adults concluded that vitamin D supplementation does not influence BMD or fracture risk when baseline 25OHD concentrations exceed 40 nmol/L.^6^ A more recent meta-analysis of individual participant data from 1439 healthy children participating in 9 RCTs of vitamin D supplementation^7^ reported a small positive effect of the intervention on total hip areal BMD but no statistically significant effects of vitamin D on total body BMC or on BMD at the femoral neck, LS, or forearm after 1 yr of supplementation. There was no clear evidence of linear or nonlinear interactions between baseline 25OHD and treatment; effects were similar in baseline 25OHD subgroups (cutoff of 35 or 50 nmol/L). However, despite evidence that relationships between vitamin D status, PTH, BMD, and fracture risk differ between children of White European vs Black African ancestry,^8–10^ RCTs to determine the effects of vitamin D on BMC and bone turnover markers in African children are lacking.

To address this deficit, we performed a sub-study nested within the ViDiKids trial, a multicenter phase 3 RCT, which investigated the effects of weekly oral administration of 10 000 IU vitamin D_3_ for 3 yr on the primary outcome of tuberculosis infection in a cohort of 1682 schoolchildren aged 6–11 yr living in a socio-economically disadvantaged peri-urban district of Cape Town, South Africa.^11^ Sub-study outcomes were BMC at the whole body less head (WBLH) and LS sites, and serum concentrations of 25(OH)D_3_, PTH, alkaline phosphatase (ALP), CTX, and P1NP. The influence of vitamin D supplementation on incidence of fractures in the study population as a whole was also investigated.

## Materials and methods

### Trial design, setting, approvals, and registration

We conducted a multicenter phase 3 double-blind individually randomized placebo-controlled trial in 23 government schools in Cape Town, South Africa, as previously described.^11^ The primary outcome was the acquisition of latent tuberculosis infection; the current manuscript reports the effects of the intervention on pre-specified secondary outcomes relating to fracture incidence in all study participants, and BMC and serum concentrations of 25(OH)D_3_, adjusted calcium, PTH and markers of bone turnover in a subset of participants who additionally took part in a nested bone sub-study. The trial was sponsored by Queen Mary University of London, approved by the University of Cape Town Faculty of Health Sciences Human Research Ethics Committee (Ref: 796/2015) and the London School of Hygiene and Tropical Medicine Observational/Interventions Research Ethics Committee (Ref: 7450-2) and registered on the South African National Clinical Trials Register (DOH-27-0916-5527) and ClinicalTrials.gov (ref NCT02880982).

### Participants

Inclusion criteria for the main trial were enrollment in grades 1–4 at a participating school; age 6 to 11 yr at screening; and written informed assent / consent to participate in the main trial provided by children and their parent / legal guardian, respectively. Exclusion criteria for the main trial were a history of previous latent TB infection, active TB disease or any chronic illness other than asthma (including known or suspected HIV infection) prior to enrollment; use of any regular medication other than asthma medication; use of vitamin D supplements at a dose of more than 400 IU/d in the month before enrollment; plans to move away from study area within 3 yr of enrollment; inability to swallow a placebo soft gel capsule with ease; and clinical evidence of rickets or a positive QuantiFERON-TB Gold Plus (QFT-Plus) assay result at screening. An additional inclusion criterion for the bone sub-study was enrollment in grade 4 at a participating school.

### Enrollment

Parents or legal guardians were invited to provide written informed consent for their child to participate in the main trial during a home visit, unless their child was eligible for the bone sub-study, in which case they were invited to provide written informed consent for their child to participate in both the main trial and the bone sub-study until a total of 450 sub-study participants were randomized. If parents / legal guardians consented, they were asked to provide details of their child’s dietary intake of foods containing vitamin D and calcium in the previous month, which were captured on an electronic case report form (Figure S1, Supplemental Material). Their children were then invited to provide written assent to participate in the main trial +/− the bone sub-study (if eligible) at a school-based visit. If they agreed, a clinically trained member of the study team screened them for symptoms and signs of rickets. For all participants, a blood sample was taken for a QFT-Plus assay and separation and storage of serum for determination of 25OHD concentrations as described below. For bone sub-study participants, additional blood was taken for the determination of serum concentrations of calcium, albumin, PTH, total ALP, P1NP, and CTX as described below. Participants were reviewed when baseline QFT-Plus results were available. Those with a positive QFT-Plus result were excluded from the trial and screened for active TB. Those with an indeterminate QFT-Plus result were excluded from the trial without screening for active TB. Those with a negative QFT-Plus result were deemed eligible to participate and underwent measurement of weight (using a digital floor scale, Charder Medical) and height (using a portable HM200P stadiometer, Charder Medical). Bone sub-study participants also underwent baseline DXA scanning as described below.

### Randomization and blinding

Full details of randomization and blinding procedures have been described previously^11^ and are presented in Supplemental Material. Briefly, eligible and assenting children whose parents consented to their participation in the trial were individually randomized to receive a weekly capsule containing vitamin D_3_ or placebo for 3 yr, with a one-to-one allocation ratio and randomization stratified by school of attendance. Treatment allocation was concealed from participants, care providers, and all trial staff (including senior investigators and those assessing outcomes) until completion of the trial to maintain the double-blind.

### Intervention

Study medication comprised a 3-yr course of weekly soft gel capsules manufactured by the Tishcon Corporation, containing either 0.25 mg (10 000 international units) cholecalciferol (vitamin D_3_) in olive oil (intervention arm) or olive oil without any vitamin D_3_ content (placebo arm). Active and placebo capsules had identical appearance and taste. Capsules were taken under direct observation of study staff during school termtime. During summer holidays (8 wk), packs containing 8 doses of study medication were provided for administration by parents, together with a participant diary. Following shorter school holidays (≤4 wk), and/or if participants missed 1 or more doses of study medication during term time, up to 4 “catch-up” doses were administered at the first weekly visit attended following the missed dose(s). During the initial national lockdown for COVID-19 in South Africa (27March to 1 May, 2020), participants did not receive any study medication. During subsequent school closures due to coronavirus disease (COVID-19), 2 rounds of 8-wk holiday packs were provided to participants: these were sufficient to cover their requirements until schools re-opened. At weekly study visits during school terms, the study team captured data on adverse events and supervised the administration of study capsules. At 1-yr, 2-yr, and 3-yr follow-up, history of fractures in the previous year was captured using an electronic case report form (Figure S2, Supplemental Material). At 3-yr follow-up, all participants were invited to provide a blood sample for QFT-Plus testing and separation and storage of serum for determination of 25(OH)D_3_ concentrations. Bone sub-study participants were invited to give extra blood for determination of end-study serum concentrations of calcium, albumin, PTH, total ALP, P1NP, and CTX as described below, and to undergo repeat DXA scanning as at baseline.

### Outcomes

The primary outcome for the main trial, reported elsewhere,^11^ was the QFT-Plus result at the manufacturer-recommended 0.35 IU/mL threshold at the end of the study. Pre-specified outcomes for the bone sub-study were BMC at the WBLH and LS sites and serum concentrations of 25(OH)D_3_, adjusted calcium, PTH, ALP, CTX, and P1NP at 3-yr follow-up. Fracture incidence was a pre-specified secondary outcome for the main trial. LS BMD, LS bone mineral apparent density (BMAD), and height-for-age z-scores were analyzed as exploratory secondary outcomes in response to reviewer requests.

### Dual energy X-ray Absorptiometry

DXA scans were performed at the Sports Science Institute of South Africa, University of Cape Town, by a trained radiographer on one Hologic bone densitometer (Discovery-W®, Hologic) using standard procedures and analyzed using Apex software (Version 13.4.1). Quality assurance checks were carried out prior to scanning and generated coefficients of variation <0.5%. WBLH and LS scans were performed to measure BMC with and without volumetric correction and correction for bone area, height, and weight as described elsewhere.^12^

### Laboratory assessments

Biochemical analyses were performed at the Bioanalytical Facility, University of East Anglia according to the manufacturers’ instructions and under Good Clinical and Laboratory Practice conditions. Serum concentrations of 25(OH)D_3_ and 25(OH)D_2_ were measured using liquid chromatography tandem mass spectrometry as previously described.^13^ 25(OH)D_2_ was undetectable in all samples. 25(OH)D_3_ was calibrated using standard reference material SRM972a from the National Institute of Science and Technology (NIST), and the assay showed linearity between 0 and 200 nmol/L. The inter/intra-assay coefficient of variation (CV) across the assay range was ≤9%, and the lower limit of quantification was 0.1 nmol/L. The assay showed <6% accuracy bias against NIST reference method on the vitamin D external quality assessment scheme (http://www.deqas.org/; accessed on 30 November, 2022). Serum concentrations of total calcium, albumin, and creatinine were measured by spectrophotometric methods on the Cobas c501 platform (Roche Diagnostics) according to the manufacturer’s instructions. The inter-assay CV for total calcium and albumin was ≤ 2.1% across the assay working ranges of 0.2 to 7.5 mmol/L and 2 to 60 g/L. Albumin-adjusted calcium was calculated as total calcium (mmol/l) + 0.02 × (40 – albumin [g/l]). Serum ALP concentrations were measured by colorimetric assay on the Cobas e501 platform (Roche): the inter-assay CV across the assay working range of 5–1200 U/L was ≤2.4%. Serum concentrations of CTX, PINP, PTH, and total ALP were measured using electrochemiluminesence immunoassays performed on the Cobas e601 platform (Roche). The inter-assay CV for CTX was ≤3% between 0.2 and 1.5 μg/L with a sensitivity of 0.01 μg/L. The inter-assay CV for P1NP was ≤3% between 20 and 600 μg/L with a sensitivity of 8 μg/L. The inter-assay CV for PTH was ≤3.8% between 0.127 and 530 pmoL/L. QFT-Plus assays were performed by the Bio Analytical Research Corporation South Africa according to the manufacturer’s instructions.

### Sample size

Sample size for the main trial was predicated on power to detect an effect of the intervention on the primary outcome (the proportion of children with a positive QFT-Plus assay result at 3-yr follow-up), as previously described.^11^ The bone sub-study was powered to detect a clinically significant effect of vitamin D on BMC: assuming 29% loss to follow-up at 3 yr, we calculated that enrollment of 450 participants would provide 88% power to detect a difference of 0.35 SDs between arms for mean BMC at either site investigated at the 5% significance level.

### Statistical analyses

Statistical analyses were performed using Stata software (Version 17.0; StataCorp) according to intention to treat. LS BMAD was calculated by dividing LS BMC by LS bone area.^1,5^ Data on participants’ age, sex, and height were used to compute height-for-age z-scores based on WHO 2007 growth reference data for 5–19 yr as previously reported.^14^ Effects of allocation to vitamin D vs placebo on BMC and other continuous outcomes were estimated using mixed-effects linear regression with adjustment for baseline value of the outcome measure and a random effect of school of attendance, with results reported as adjusted mean differences (aMDs) with 95% CIs. Pre-specified sub-group analyses were conducted to determine whether the effect of vitamin D supplementation was modified by sex (male vs female), baseline deseasonalized 25(OH)D_3_ concentration, calculated using a sinusoidal model as previously described^15^ (<75 vs ≥75 nmol/L), and estimated daily calcium intake (< vs ≥ median value of 466 mg/d, calculated as described in Supplemental Material, Table S1). These were performed by repeating efficacy analyses with the inclusion of an interaction term between allocation (to vitamin D vs placebo) and each posited effect-modifier with presentation of the *P*-value associated with this interaction term. Given the number of potential effect modifiers and secondary outcome measures, these analyses are considered exploratory. Analysis of fracture incidence was modified to reflect different data structure, ie multiple observations per individual where outcome was the reporting of a fracture (0 = no, 1 = yes) at each yearly assessment. Effects of treatment on the proportion of participants reporting 1 or more fractures per year were estimated by fitting allocation (vitamin D vs placebo) as the sole fixed effect in a mixed effects logistic regression model with random effect terms included for individual and school of attendance to allow for potential clustering at these levels. Results are reported as odds ratios with 95% CIs. Interim safety assessments, where Independent Data Monitoring Committee (IDMC) members reviewed accumulating serious adverse event data, were performed at 6-monthly intervals. At each review, the IDMC recommended continuation of the trial. No interim efficacy analysis was performed.

## Results

### Participants

**Figure 1:**
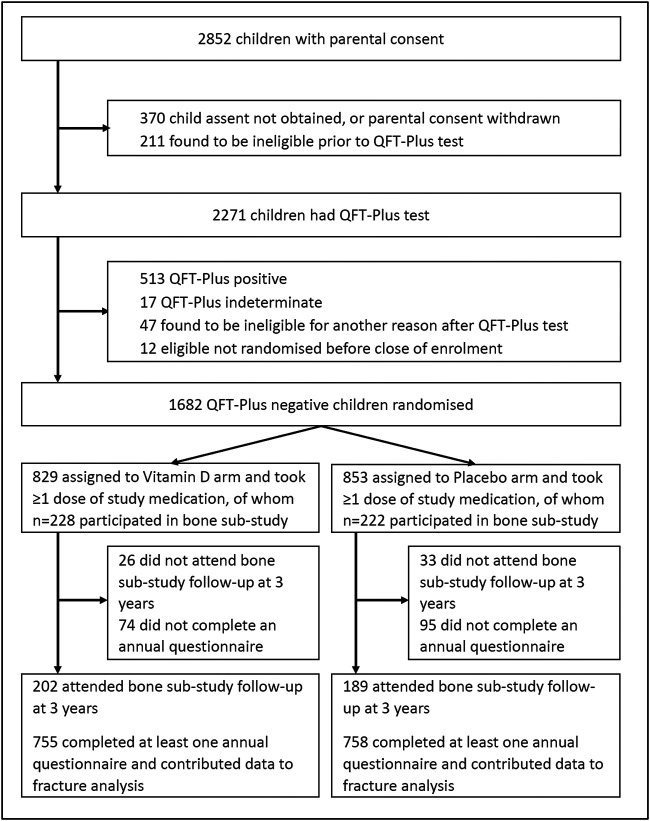
Participant flow.

Of 2852 children screened for eligibility from March 2017 to March 2019, 2271 underwent QFT-Plus testing. About 1682 (74.1%) of these tested negative and were randomly assigned to receive vitamin D_3_ (829 participants) or placebo (853 participants) as previously described^11^; 450/1682 (26.8%) participants in the main trial also participated in the bone sub-study, of whom 228 vs 222 participants were allocated to the vitamin D vs placebo arms, respectively (Figure 1). Table 1 presents baseline characteristics of children in the main trial and in the bone sub-study, overall and by study arm. Mean age was higher among participants in the sub-study vs all those in the main trial (10.1 vs 8.9 yr, respectively), reflecting the fact that participation in the sub-study was restricted to children enrolled in grade 4. Baseline characteristics were otherwise well balanced for all participants in the main trial vs those who additionally participated in the sub-study: 52.4% vs 52.0% were female and mean serum 25(OH)D_3_ concentrations were 71.2 nmol/L vs 70.0 nmol/L, respectively. Within the main trial and the sub-study, baseline characteristics of those randomized to vitamin D vs placebo were also well balanced.

**Table 1:** Participants’ baseline characteristics by allocation: bone sub-study and fracture study.

		**Bone sub-study** **(*n* = 450 subset)**			**Fracture study** **(*n* = 1682)**		
		**Overall (*n* = 450)**	**Vitamin D arm (*n* = 228)**	**Placebo arm (*n* = 222)**	**Overall (*n* = 1682)**	**Vitamin D arm (*n* = 829)**	**Placebo arm (*n* = 853)**
Mean age, years (SD)		10.1 (0.7)	10.2 (0.7)	10.0 (0.6)	8.9 (1.4)	8.9 (1.4)	8.8 (1.3)
Female sex, *n* (%)		234 (52.0)	116 (50.9)	118 (53.2)	880 (52.4)	437 (52.8)	443 (51.9)
Ethnic origin^a^	Xhosa, *n* (%)	424 (96.4)	214 (96.0)	210 (96.8)	1615 (97.9)	788 (97.3)	827 (98.5)
	Other, *n* (%)	16 (3.6)	9 (4.0)	7 (3.2)	35 (2.1)	22 (2.7)	13 (1.5)
Type of residence	Brick, *n* (%)	230 (51.1)	121 (53.1)	109 (49.1)	867 (51.5)	423 (51.0)	444 (52.1)
	Informal, *n* (%)	220 (48.9)	107 (46.9)	113 (50.9)	815 (48.5)	406 (49.0)	409 (47.9)
Parental education^a,b^	Primary school, *n* (%)	24 (5.3)	16 (7.0)	8 (3.6)	60 (3.6)	34 (4.1)	26 (3.1)
	Secondary school or higher, *n* (%)	426 (94.7)	212 (93.0)	214 (96.4)	1618 (96.4)	792 (95.9)	826 (96.9)
Mean monthly household income, 1000 ZAR (SD)		1.6 (2.3)	1.5 (2.7)	1.6 (1.9)	1.9 (2.2)	1.8 (2.1)	2.0 (2.2)
Mean BMI-for-age z-score (SD)^a^		0.2 (1.0)	0.2 (1.0)	0.2 (1.0)	0.3 (1.1)	0.3 (1.1)	0.3 (1.0)
Mean height-for-age z-score (SD)^a^		−0.4 (1.0)	−0.4 (1.0)	−0.4 (0.9)	−0.6 (1.2)	−0.6 (1.3)	−0.5 (1.1)
Calcium intake^a^	≤300 mg/d, *n* (%)	67 (15.2)	32 (14.4)	35 (16.0)	239 (14.6)	104 (12.9)	135 (16.3)
	>300 & ≤500 mg/d, *n* (%)-	208 (47.2)	98 (44.1)	110 (50.2)	672 (41.1)	328 (40.8)	344 (41.4)
	>500 mg/d, *n* (%)	166 (37.6)	92 (41.4)	74 (33.8)	723 (44.2)	372 (46.3)	351 (42.3)
Mean serum 25(OH)D_3_ concentration, nmol/L (SD)^a,c^		70.0 (13.5)	70.4 (12.1)	69.6 (14.9)	71.2 (14.8)	71.2 (14.5)	71.1 (15.0)
Serum 25(OH)D_3_ concentration, category^a,c^	<25 nmol/L, *n* (%)	1 (0.3)	0 (0)	1 (0.6)	1 (0.1)	0 (0.0)	1 (0.1)
	≥ 25 & < 50 nmol/L, *n* (%)	20 (5.5)	7 (3.7)	13 (7.4)	74 (5.4)	34 (5.1)	40 (5.8)
	≥ 50 & < 75 nmol/L, *n* (%)	214 (59.0)	114 (61.0)	100 (56.8)	787 (57.7)	394 (58.8)	393 (56.6)
	≥75 nmol/L, *n* (%)	128 (35.3)	66 (35.3)	62 (35.2)	502 (36.8)	242 (36.1)	260 (37.5)
Mean BMC at WBLH, g (SD)^a^		759.3 (133.8)	772.4 (137.1)	745.9 (129.1)			
Mean BMC at LS, g (SD)^a^		23.7 (4.6)	24.3 (4.8)	23.2 (4.3)			
Mean BMD at LS, g/cm^2^ (SD)		0.59 (0.08)	0.60 (.09)	0.58 (0.08)			
Mean BMAD at LS, g/cm^3^ (SD)		0.09 (0.01)	0.09 (0.01)	0.09 (0.01)			
Mean serum adjusted calcium concentration, mmol/L (SD)^a^		2.38 (0.06)	2.38 (0.06)	2.38 (0.06)			
Mean PTH concentration, pmol/L (SD)^a^		3.68 (1.43)	3.79 (1.56)	3.56 (1.28)			
Serum PTH concentration, category	<1.6 pmol/L, *n* (%)	13 (3.6%)	3 (1.6%)	10 (5.7%)			
	≥1.6 & <6.9 pmol/L, *n* (%)	336 (93.6%)	173 (94.5%)	163 (92.6%)			
	≥6.9 pmol/L, *n* (%)	10 (2.8%)	7 (3.8%)	3 (1.7%)			
Mean total ALP, IU/L (SD)^a^		282.02 (75.58)	281.02 (72.40)	283.08 (78.98)			
Mean CTX concentration, μg/L (SD)^a^		1.20 (0.41)	1.18 (0.41)	1.23 (0.41)			
Mean P1NP concentration, μg/L (SD)^a^		803.85 (265.32)	814.92 (280.44)	792.21 (248.72)			

ZAR, South African Rand.

a Missing data (for bone sub-study: ethnicity, *n* = 5 vitamin D arm, *n* = 5 placebo arm; for calcium intake, *n* = 6 Vitamin D arm, *n* = 3 placebo arm; for serum 25(OH)D_3_ concentration, *n* = 66 vitamin D arm, *n* = 62 placebo arm; for serum-adjusted calcium and total ALP concentration, *n* = 41 vitamin D arm, *n* = 45 placebo arm; for PTH: *n* = 42 vitamin D arm, *n* = 46 placebo arm; for CTX: *n* = 41 vitamin D arm, *n* = 46 placebo arm; for P1NP: *n* = 43 vitamin D arm, *n* = 46 placebo arm; for fracture study: ethnicity, *n* = 19 vitamin D arm, *n* = 13 placebo arm; parental education, *n* = 3 vitamin D arm, *n* = 1 placebo arm; BMI-for-age z-score, *n* = 2 vitamin D arm, *n* = 0 placebo arm; height-for-age z-score, *n* = 2 vitamin D arm, *n* = 0 placebo arm; for calcium intake, *n* = 25 Vitamin D arm, *n* = 23 placebo arm; for serum 25(OH)D_3_ concentration, *n* = 159 vitamin D arm, *n* = 159 placebo arm)

^b^Highest level of education of at least 1 parent.

^c^Deseasonalized values.

The median duration of follow-up was 3.16 yr (interquartile range, 2.83 to 3.38 yr) and was not different between the 2 study arms. For the main trial, mean serum 25(OH)D_3_ concentrations at 3-yr follow-up were higher among children randomized to receive vitamin D vs placebo (104.3 vs 64.7 nmol/L, respectively; mean difference 39.7 nmol/L, 95% CI for difference 37.6 to 41.9 nmol/L).

### Bone mineral content

Table 2 presents values for mean BMC at the WBLH and LS sites at 3-yr follow-up by allocation. No difference in either outcome was seen between participants randomized to vitamin D vs placebo overall (for WBLH: 1112.9 vs 1071.5 g respectively, aMD −8.0, 95% CI, −30.7 to 14.7, *P* = .49; for LS: 36.2 vs 34.2 respectively, aMD −0.3, 95% CI, −1.3 to 0.8, *P* = .65). Sub-group analysis by sex, baseline 25(OH)D_3_ concentration, and calcium intake did not reveal evidence of effect modification by any of these factors (*P*-values for interaction ≥.11). Overall results were also null when statistical analyses were conducted with volumetric correction and correction for bone area, height, and weight (Table S2, Supplemental Material). Exploratory analyses to determine the influence of vitamin D on LS BMD, LS BMAD, and height-for-age z-score, overall and by sub-group, also yielded null results (Tables S3–S5, Supplemental Material).

**Table 2:** Uncorrected^a^ end-trial bone mineral content at the whole body less head and LS sites by allocation: overall and by sub-groups.

		**Vitamin D arm: mean value, g (SD) [N]**	**Placebo arm: mean value, g (SD) [N]**	**Adjusted mean difference (95% CI)** ^**b**^	***P***	***P* for interaction**
**Whole body less head**						
Overall		1112.9 (255.3) [202]	1071.5 (221.9) [189]	−8.0 (−30.7 to 14.7)	.49	—
By sex	Male	1102.3 (268.5) [97]	1031.4 (225.4) [89]	1.9 (−32.0 to 35.7)	.91	.11
	Female	1122.7 (243.4) [105]	1107.2 (213.5) [100]	−20.8 (−46.8 to 5.3)	.12	
By baseline 25(OH)D_3_ concentration^c^	<75 nmol/L	1137.0 (270.8) [103]	1099.1 (224.2) [96]	−16.5 (−47.5 to 14.5)	.30	.76
	≥75 nmol/L	1119.3 (269.6) [60]	1058.4 (215.9) [54]	−8.4 (−50.5 to 33.7)	.70	
By calcium intake	<median^d^	1111.3 (249.5) [94]	1057.8 (212.9) [97]	5.2 (−27.6 to 38.1)	.75	.23
	≥median^d^	1120.7 (263.6) [102]	1094.0 (228.9) [89]	−19.3 (−50.9 to 12.3)	.23	
**LS**						
Overall		36.2 (10.1) [202]	34.2 (8.0) [189]	−0.3 (−1.3 to 0.8)	.65	
By sex	Male	33.2 (9.7) [97]	31.1 (6.8) [89]	0.2 (−1.2 to 1.5)	.81	.28
	Female	39.0 (9.7) [105]	37.0 (8.0) [100]	−0.5 (−1.7 to 0.7)	.40	
By baseline 25(OH)D_3_ concentration^c^	<75 nmol/L	37.5 (10.7) [103]	35.4 (8.4) [96]	−0.2 (−1.6 to 1.3)	.82	.82
	≥75 nmol/L	36.2 (10.5) [60]	33.1 (6.9) [54]	−0.32(−2.4 to 1.8)	.77	
By calcium intake	<median^d^	35.5 (9.9) [94]	33.9 (7.8) [97]	0.1 (−1.4 to 1.6)	.93	.54
	≥median^d^	36.9 (10.2) [102]	34.8 (8.2) [89]	−0.6 (−2.1 to 0.9)	.45	

N, number.

a That is without volumetric correction or correction for bone area, height, and weight.

^b^Adjusted for baseline value and school of attendance.

^c^Deseasonalized values.

^d^Median calcium intake 466 mg/d.

### Biochemical outcomes

Table 3 presents mean values for serum concentrations of 25(OH)D_3_, adjusted calcium, PTH, and bone turnover markers at 3-yr follow-up in bone sub-study participants by allocation. In analyses of the sub-study population as a whole, mean serum 25(OH)D_3_ concentration at 3 yr was higher among participants allocated to vitamin D vs placebo (aMD 39.9 nmol/L, 95% CI for difference 36.1 to 43.6 nmol/L, *P* < .001), and mean serum PTH concentration was lower (aMD −0.55 pmol/L, 95% CI, −0.94 to −0.17, *P* = .005). Proportions of participants with end-study serum PTH concentration above 6.9 pmol/L (the upper limit of normal) and below 1.6 pmol/L (the lower limit of normal) are presented in Table S6, Supplementary Material. No inter-arm differences in end-study serum concentrations of adjusted calcium, ALP, CTX, or P1NP were seen. Sub-group analyses indicated that effects of vitamin D were modified by baseline vitamin D status for the outcome of serum 25(OH)D_3_ concentration (*P* for interaction .04); by calcium intake for the outcome of ALP concentration (*P* for interaction .02); and by sex for the outcomes of serum CTX and P1NP concentrations (*P*-values for interaction .03 and .049, respectively). *P*-values for interaction were ≥ .10 for all other sub-group analyses of biochemical outcomes.

**Table 3:** Serum concentrations of 25(OH)D_3_, adjusted calcium, PTH, and bone turnover markers at 3-yr follow-up by allocation: bone sub-study, overall, and by sub-group.

		**Vitamin D arm: mean value (SD) [N]**	**Placebo arm: mean value (SD) [N]**	**Adjusted mean difference (95% CI)** ^**a**^	***P***	***P* for interaction**
**25(OH)D**_**3**_ **, nmol/L**						
Overall		97.6 (21.5) [202]	58.8 (14.2) [187]	39.9 (36.1 to 43.6)	<.001	**—**
By sex	Male	99.8 (22.9) [97]	62.7 (13.5) [88]	39.7 (33.9 to 45.4)	<.001	.89
	Female	95.6 (20.0) [105]	55.3 (14.1) [99]	40.1 (35.5 to 44.7)	<.001	
By baseline 25(OH)D_3_ concentration^b^	<75 nmol/L	98.8 (20.6) [94]	57.9 (14.4) [95]	43.3 (38.2 to 48.3)	<.001	.04
	≥75 nmol/L	95.7 (20.5) [102]	59. 8 (14.2) [89]	35.6 (30.4 to 40.7)	<.001	
By calcium intake	<Median^c^	92.4 (19.3) [103]	53.7 (13.8) [94]	37.7 (33.2 to 42.2)	<.001	.11
	≥Median^c^	107.9 (22.8) [60]	65.0 (12.3) [54]	44.3 (37.7 to 50.9)	<.001	
**Adjusted calcium, mmol/L**						
Overall		2.28 (0.07) [201]	2.27 (0.07) [186]	0.01 (0.00 to 0.03)	.06	
By sex	Male	2.28 (0.07) [97]	2.27 (0.07) [88]	0.02 (0.00 to 0.04)	.12	.59
	Female	2.27 (0.07) [104]	2.27 (0.06) [98]	0.01 (−0.01 to 0.03)	.32	
By baseline 25(OH)D_3_ concentration^b^	<75 nmol/L	2.28 (0.07) [94]	2.27 (0.07) [95]	0.01 (−0.01 to 0.03)	.34	.70
	≥75 nmol/L	2.27 (0.07) [101]	2.27 (0.06) [88]	0.02 (0.00 to 0.04)	.08	
By calcium intake	<Median^c^	2.28 (0.07) [103]	2.27 (0.07) [94]	0.01 (−0.01 to 0.03)	.22	.64
	≥Median^c^	2.29 (0.08) [59]	2.27 (0.06) [54]	0.02 (−0.01 to 0.04)	.12	
**PTH, pmol/L**						
Overall		4.18 (1.77) [201]	4.51 (1.86) [186]	−0.55 (−0.94 to −0.17)	.005	
By sex	Male	4.14 (1.75) [97]	4.20 (1.74) [88]	−0.22 (−0.76 to 0.32)	.43	.10
	Female	4.23 (1.80) [104]	4.78 (1.92) [98]	−0.83 (−1.38 to −0.29)	.003	
By baseline 25(OH)D_3_ concentration^b^	<75 nmol/L	4.43 (1.79) [103]	4.92 (1.96) [94]	−0.57 (−1.07 to −0.06)	.03	.73
	≥75 nmol/L	3.93 (1.95) [59]	4.21 (1.63) [54]	−0.52 (−1.08 to 0.04)	.07	
By calcium intake	<Median^c^	4.22 (1.84) [94]	4.38 (1.80) [95]	−0.48 (−1.05 to 0.09)	.10	.74
	≥Median^c^	4.17 (1.72) [101]	4.63 (1.95) [88]	−0.58 (−1.13 to −0.04)	.04	
**Total ALP, IU/L**						
Overall		303.5 (111.6) [202]	302.5 (116.9) [186]	−5.2 (−30.8 to 20.5)	.69	
By sex	Male	361.9 (93.4) [97]	349.2 (120.4) [88]	0.4 (−30.7 to 31.6)	.98	.37
	Female	249.5 (99.6) [105]	260.6 (96.5) [98]	−17.4 (−46.0 to 11.3)	.23	
By baseline 25(OH)D_3_ concentration^b^	<75 nmol/L	284.1 (107.6) [103]	305.1 (132.6) [94]	−19.2 (−52.5 to 14.2)	.26	.18
	≥75 nmol/L	323.3 (117.0) [60]	300.7 (99.5) [54]	10.3 (−26.9 to 47.5)	.59	
By calcium intake	<Median^c^	328.5 (116.4) [94]	295.4 (103.2) [95]	27.2 (−7.9 to 62.3)	.13	.02
	≥Median^c^	280.5 (103.9) [102]	310.0 (131.2) [88]	−32.4 (−70.2 to 5.5)	.09	
**CTX, μg/L**						
Overall		1.30 (0.72) [201]	1.21 (0.49) [186]	0.07 (−0.07 to 0.21)	.31	
By sex	Male	1.62 (0.80) [97]	1.39 (0.43) [88]	0.19 (0.00 to 0.38)	.048	.03
	Female	0.99 (0.48) [104]	1.05 (0.48) [98]	−0.09 (−0.23 to 0.06)	.23	
By baseline 25(OH)D_3_ concentration^b^	<75 nmol/L	1.24 (0.70) [103]	1.18 (0.50) [94]	0.07 (−0.09 to 0.24)	.39	.98
	≥75 nmol/L	1.34 (0.77) [59]	1.28 (0.52) [54]	0.06 (−0.18 to 0.30)	.61	
By calcium intake	<Median^c^	1.37 (0.77) [94]	1.27 (0.45) [95]	0.04 (−0.15 to 0.23)	..69	.53
	≥Median^c^	1.22 (0.69) [101]	1.14 (0.52) [88]	0.11 (−0.08 to 0.31)	.26	
**P1NP, μg/L**						
Overall		726.5 (408.7) [201]	692.8 (334.9) [186]	46.2 (−38.2 to 130.5)	.28	
By sex	Boys	928.8 (418.8) [97]	816.5 (315.1) [88]	106.6 (−8.8 to 222.1)	.07	.049
	Girls	537.9 (294.1) [104]	581.8 (313.8) [98]	−19.1 (−104.3 to 66.1)	.66	
By baseline 25(OH)D_3_ concentration^b^	<75 nmol/L	665.3 (362.8) [103]	658.3 (361.5) [94]	10.7 (−89.1 to 110.6)	.83	.26
	≥75 nmol/L	795.6 (472.4) [59]	690.4 (309.9) [54]	101.2 (−46.0 to 248.4)	.18	
By calcium intake	<Median^c^	800.2 (447.6) [94]	699.0 (307.6) [95]	109.8 (−17.1 to 236.6)	.09	.16
	≥Median^c^	659.2 (365.8) [101]	678.6 (362.3) [88]	−4.7 (−116.7 to 107.3)	.93	

N, number.

a Adjusted for baseline value and school of attendance.

^b^Deseasonalized values.

^c^Median calcium intake 466 mg/d.

### Fractures

Seventeen participants reported 17 fractures during follow-up (11 upper limb, 4 lower limb and 2 at another anatomical site; Table S7, Supplemental Material). Allocation to vitamin D vs placebo did not influence the proportion of participants reporting 1 or more fractures (adjusted odds ratio [aOR] 0.70, 95% CI, 0.27 to 1.85, *P* = .48; Table 4). Only 2 fractures were associated with high trauma; both occurred in participants assigned to placebo. A sensitivity analysis excluding these 2 events yielded a similar effect estimate to the primary analysis (data not shown). Sub-group analyses evaluating the effects of the intervention by sex, baseline serum 25(OH)D_3_ concentration, and calcium intake revealed no evidence of effect modification (*P*-values for interaction ≥.77 where calculable). Similarly null results were obtained for fractures reported as being X-ray-confirmed and for those reported as being treated with a plaster cast (Tables S8 and S9, Supplemental Material).

**Table 4:** Proportion reporting one or more fractures by follow-up time point and allocation, overall and by sub-group.

		**Time point**	**Vitamin D arm (%)**	**Placebo arm (%)**	**Adjusted odds ratio (95% CI)** ^**a**^	***P***	***P* for interaction** ^**b**^
Overall		1 yr	1/671 (0.15)	1/668 (0.15)	0.70 (0.27–1.85)	.48	—
		2 yr	2/614 (0.33)	5/606 (0.83)			
		3 yr	4/669 (0.60)	4/689 (0.58)			
By sex	Male	1 yr	0/307 (0.00)	1/312 (0.32)	0.73 (0.26–2.11)	.56	.84
		2 yr	2/284 (0.70)	4/284 (1.41)			
		3 yr	4/309 (1.29)	4/325 (1.23)			
	Female	1 yr	1/362 (0.28)	0/356 (0.00)	0.98 (0.06–15.76)	.99	
		2 yr	0/328 (0.00)	1/322 (0.31)			
		3 yr	0/358 (0.00)	0/364 (0.00)			
By baseline 25(OH)D_3_ concentration^c^	<75 nmol/L	1 yr	0/353 (0.00)	0/335 (0.00)	^d^	^d^	^d^
		2 yr	0/326 (0.00)	3/323 (0.93)			
		3 yr	0/340 (0.00)	1/358 (0.28)			
	≥75 nmol/L	1 yr	0/189 (0.00)	1/203 (0.49)	0.84 (0.17–3.92)	>.99	
		2 yr	2/172 (1.16)	2/178 (1.12)			
		3 yr	2/197 (1.02)	2/205 (0.98)			
By calcium intake	<Median^e^	1 yr	0/316 (0.00)	1/335 (0.30)	0.64 (0.19–2.19)	.47	.77
		2 yr	2/287 (0.70)	2/308 (0.65)			
		3 yr	2/313 (0.64)	4/361 (1.11)			
	≥Median^e^	1 yr	1/332 (0.30)	0/317 (0.00)	0.90 (0.18–4.51)	.90	
		2 yr	0/309 (0.00)	3/281 (1.07)			
		3 yr	2/336 (0.60)	0/310 (0.00)			

a Adjusted for random effects of school and individual.

^b^*P*-value for treatment-by-subgroup interaction.

^c^Deseasonalized values.

^d^Not calculated due to absence of events in vitamin D arm participants with baseline 25(OH)D_3_ < 75 nmol/L. ^e^Median calcium intake 466 mg/d.

### Adverse events

Incidence of adverse events by trial arm has been reported elsewhere.^11^ No serious events arising in the trial were adjudged related to administration of vitamin D or placebo. No hypercalciuria was observed in the 200 participants assigned to a safety sub-study, as monitored at 6, 12, 24, and 36 mo post-randomization, as reported elsewhere.^11^ No participant had 25(OH)D_3_ concentration > 220 nmol/L at any follow-up timepoint, as reported elsewhere.^11^ A total of 9 sub-study participants (5 vs 4 randomized to vitamin D vs placebo, respectively) had end-study serum PTH concentrations less than 1.6 pmol/L (the lower limit of normal).

## Discussion

We report findings of the first RCT to investigate the effects of vitamin D supplementation on BMC in the children of Black African ancestry. Vitamin D insufficiency (25OHD 50–74.9 nmol/L) was common at baseline, and weekly oral administration of 10 000 IU vitamin D_3_ for 3 yr was effective in suppressing serum PTH concentrations and elevating 25OHD concentrations above the 75 nmol/L threshold. However, these biochemical effects were not associated with changes in BMC or serum concentrations of bone turnover markers in the study population as a whole. No evidence for effect modification by baseline vitamin D status, sex, or calcium intake was found. Neither was any effect of the intervention seen on incidence of fractures.

Null results of our trial for BMC outcomes are in accordance with a prior expert consensus statement,^5^ but contrast with positive findings from other studies that have investigated the effects of higher dose vitamin D on BMC. Fuleihan and colleagues reported that weekly administration of 14 000 IU vitamin D for 1 yr increased bone area and total hip BMC in HIV-uninfected girls aged 10–17 yr in Lebanon; sub-group analysis revealed that these changes were restricted to premenarcheal participants.^16^ Meta-analysis of RCT investigating the effects of higher-dose vitamin D (1600 to 4000 IU/d) in HIV-infected adolescents and young adults living in the USA^17^ and Thailand^18^ also revealed vitamin D-induced increases in total BMC.^19^ Differences in outcomes between these studies vs our own might reflect the relatively high baseline vitamin D status among participants in our study. This factor might also explain why we saw no effect of the intervention on bone turnover markers. However, results from our linked trial in Mongolian schoolchildren,^20^ whose baseline vitamin D status was much lower than in the current study, were also null for BMC outcomes. Other potential explanations for our null findings for BMC outcomes include participants’ low calcium intakes, and (for comparison with results from USA/Thailand) the fact that our participants were not HIV-infected or taking anti-retroviral therapy—both factors that may modify the effects of vitamin D supplements on BMC.^21^

Our study has several strengths. The placebo-controlled RCT design minimizes potential for observer bias and confounding to operate. Administration of vitamin D supplements was sustained (3-yr duration) and directly observed during term-time, and the dose administered was sufficient to elevate serum 25OHD concentrations into the physiological range (75–200 nmol/L) and to suppress PTH concentrations. We employed DXA, the gold standard methodology, to measure BMC, and complemented it with the measurement of markers of bone formation and resorption. The bone sub-study was large, and loss to follow-up was lower than anticipated in the power calculation: accordingly, we were well powered to detect even modest effects of the intervention on BMC. Participants were less than 12 yr old at enrollment and were therefore exposed to the intervention before the period of peak bone mineral mass accretion, thereby maximizing potential for the intervention to impact BMC and fracture risk. External validity was maximized by inclusion of both males and females.

Our study also has some limitations. Very few fractures were reported, which limited our power to detect an effect of the intervention on this outcome. Low fracture risk among participants in the current trial contrasts with the much higher event rate seen in our linked trial in Mongolia,^20^ consistent with reports that age-standardized fracture incidence in Southern Africa is among the lowest globally.^22^ Genotyping was not performed, so we were unable to test whether polymorphisms in the vitamin D receptor modified the effect of vitamin D supplementation on bone mineralization, as has been reported by others.^23^ Vitamin D deficiency (25OHD < 50 nmol/L) was uncommon at baseline, so our findings are not generalizable to populations with low baseline vitamin D status. We only investigated one vitamin D dosing regimen, without concomitant administration of calcium; our findings cannot therefore shed light on the question of whether higher or lower doses of vitamin D, with or without additional calcium supplementation, may impact BMC. It is also possible that the effects of daily administration of vitamin D may differ from those of weekly administration. Our estimates of calcium intake are approximate, due to the lack of food frequency questionnaires that have been validated for quantitation of calcium intake in the study population. The study was not formally powered for interaction analyses; accordingly, our power to detect sub-group effects was limited.

In conclusion, we report that oral administration of vitamin D at a dose of 10 000 IU/week for 3 yr was effective in elevating vitamin D status and suppressing serum PTH concentrations in HIV-uninfected Black South African schoolchildren aged 10–11 yr with a low prevalence of vitamin D deficiency at baseline. However, these effects were not associated with changes in BMC or serum concentrations of bone turnover markers. Fracture incidence was low, limiting power to detect an effect of vitamin D on this outcome.

## Supplementary Material

ViDiKids_CT_bone_suppl_26OCT23_clean_zjae007

## Data Availability

Anonymized data may be requested from the corresponding author to be shared subject to terms of research ethics committee approval.

## Author contibutions

Adrian R. Martineau conceived the study. Keren Middlekoop (equal contribution), Lisa K. Micklesfield (equal contribution), Anna K. Coussens, JN, Cyrus Cooper, Nicholas C. Harvey, Richard L. Hooper, Robert J. Wilkinson, Linda-Gail Bekker and Adrian R. Martineau contributed to study design and protocol development. Keren Middlekoop (equal contribution) led on trial implementation, with support from Justine Stewart, Carmen Delport, David A. Jolliffe, James Nuttall, Linda-Gail Bekker, and Adrian R. Martineau. Lisa K. Micklesfield (equal contribution) and Amy E. Mendham oversaw performance of DXA scans. Averalda van Graan advised on estimation of calcium intake. Jonathan C.Y. Tang and William D. Fraser performed and supervised the conduct of biochemical assays. Neil Walker, Richard L. Hooper, and Adrian R. Martineau drafted the statistical analysis plan. David A. Jolliffe, Keren Middlekoop (equal contribution), Justine Stewart, Neil Walker, and Carmen Delport managed data. Neil Walker and Keren Middlekoop (equal contribution) accessed, verified and analyzed the data underlying the study. Adrian R. Martineau wrote the first draft of the trial report. All authors made substantive comments thereon and approved the final version for submission.
